# Response to: Letter to the editor concerning "Effects of Levetiracetam and Lacosamide on survival and seizure control in 2IDH-wild type glioblastoma during temozolomide plus radiation adjuvant therapy" by Andrea Bianconi et al.

**DOI:** 10.1016/j.bas.2024.103925

**Published:** 2024-10-10

**Authors:** Andrea Bianconi, Pietro Zeppa, Diego Garbossa, Fabio Cofano

**Affiliations:** Neurosurgery Unit, “Città della Salute e della Scienza” University Hospital, Department of Neuroscience “Rita Levi Montalcini”, University of Turin, 10124, Turin, Italy

**Keywords:** Glioma, Glioblastoma, Brain tumor related epilepsy, Seizure, Anti seizure medications

Dear editor

We thank Dr. Kandaswamy and Dr. Guru for their knowledgeable comments on our study. However, we must note that most of their criticisms have been extensively discussed both in the Discussion section and in the specific Limitations paragraph (Bianconi et al., 2023).

The study is retrospective; it is not a prospective, controlled, and randomized clinical trial, nor does it aim to be one. As highlighted in the introduction, we started from preclinical and clinical evidence, particularly from Pallud et al. (2022), which suggested a possible survival benefit mediated by Levetiracetam in glioblastoma IDH-WT. We wanted to verify whether this association could be reproduced and to assess the differences with lacosamide, both in terms of survival and seizure control, considering that in our case series we had started treating patients with lacosamide in monotherapy, according to recent consensus review(Avila et al., 2023).

As for the potential selection bias among patients taking lacosamide, this is inherent in the retrospective design of the study and has been highlighted both in the limitations section and in the 'control of seizure recurrence' section. To try to limit this issue, in our cohort for the control of seizure recurrence analysis, we considered only patients in monotherapy with one ASM, thus excluding add-ons or switching between them. However, lacosamide may still have been administered in cases where the tumor could be considered more epileptogenic or the seizures more severe. This is precisely why the observed lower effectiveness of lacosamide compared to levetiracetam 'should be toned down and viewed in the context of this potential selection bias'.

The duration and dosage of ASMs were collected in the dataset, and indeed, based on Pallud's article, we tried to stratify according to the duration of treatment with levetiracetam, but without obtaining significant results. However, further dividing the sample to create additional subgroups based on duration and dosage would have fragmented the results further, making them less interpretable. Therefore, we preferred to make the sample more uniform by including patients with dosages between 1000 mg and 2500 mg daily for levetiracetam and between 100 and 200 mg daily for lacosamide, without further stratifications, although the raw data is available.

Furthermore, regarding the methylation of the MGMT promoter, while the association between MGMT methylation and the presentation with seizures is well established, it is not as straightforward that MGMT methylation is correlated with an increased risk of postoperative seizures (Feyissa et al., 2019; Pallud et al., 2024; Song et al., 2021). Although this issue is addressed in the discussion, where it is also proposed that our results could be explained by considering the response of GBM cells with hypermethylated MGMT promoters to TMZ chemotherapy. In any case, the results are not such as to question the validity of the molecular data, which are part of a large database that has already been used in the description of our case series without ever raising any doubtful results or questioning their validity in any way (Cofano et al., 2024; Specchia et al., 2021).

Regarding the lack of detailed follow-up protocols, we must strongly disagree. After surgery, patients were evaluated by the multidisciplinary neuro-oncology board and assessed every three months clinically and with contrast-enhanced MRI, and they were followed until death. If we want to identify a shortcoming, it is that the patients did not keep a seizure diary, which is not routinely done in glioma patients.

In conclusion, the retrospective design of the study provides preliminary data and can be improved by a prospective design, more detailed data collection on epileptic seizures, and randomization. However, this is the first study to compare the two drugs both in terms of seizure control and survival, and it is necessary to lay the groundwork for a possible randomized trial, as well as to confirm or refute previous evidence on the effect of levetiracetam on survival, in a cohort consisting of a considerable number of patients.

## Declaration of competing interest

The authors declare the following financial interests/personal relationships which may be considered as potential competing interests:

Andrea Bianconi reports was provided by University of Turin. If there are other authors, they declare that they have no known competing financial interests or personal relationships that could have appeared to influence the work reported in this paper.
